# A Photoelectrochemical Sensor for the Sensitive Detection of Cysteine Based on Cadmium Sulfide/Tungsten Disulfide Nanocomposites

**DOI:** 10.3390/nano14050427

**Published:** 2024-02-27

**Authors:** Yan Wang, Jiaxin Liu, Fancheng Lin

**Affiliations:** College of Chemistry, Chemical Engineering and Materials Science, Shandong Normal University, Jinan 250014, China; sdnuljx@163.com (J.L.); sdnulfc@163.com (F.L.)

**Keywords:** WS_2_ nanosheet, CdS nanoparticle, heterostructure nanocomposite, cysteine, photoelectrochemical biosensor

## Abstract

In this work, a CdS-nanoparticle-decorated WS_2_ nanosheet heterojunction was successfully prepared and first used to modify ITO electrodes for the construction of a novel photoelectrochemical sensor (CdS/WS_2_/ITO). The thin-film electrode was fabricated by combining electrophoretic deposition with successive ion layer adsorption and reaction techniques. The results indicated that the synthesized heterojunction nanomaterials displayed excellent photoelectrochemical performance which was much better than that of pristine CdS nanoparticles and 2D WS_2_ nanosheets. Owing to the formation of the surface heterojunction and the effective interfacial electric field, the enhanced separation of photogenerated electron–hole pairs led to a remarkable improvement in the photoelectrochemical activity of CdS/WS_2_/ITO. This heterojunction architecture can protect CdS against photocorrosion, resulting in a stable photocurrent. Based on the specific recognition between cysteine and CdS/WS_2_/ITO, through the specificity of Cd-S bonds, a visible-light-driven photoelectrochemical sensor was fabricated for cysteine detection. The novel photoelectrochemical biosensor exhibited outstanding analytical capabilities in detecting cysteine, with an extremely low detection limit of 5.29 nM and excellent selectivity. Hence, CdS-WS_2_ heterostructure nanocomposites are promising candidates as novel advanced photosensitive materials in the field of photoelectrochemical biosensing.

## 1. Introduction

Cysteine, a crucial amino acid rich in thiol, serves significant functions in numerous biological processes. Cysteine participates in tissue protein synthesis, post-translational modifications and the construction of active sites in certain enzymes [1]. It was found that diseases such as acquired immune deficiency syndrome, liver injury and Alzheimer’s disease are accompanied by cysteine deficiency [2]. Cysteine levels in the body have been acknowledged as a significant marker for diagnosing diseases [3]. Hence, it is crucial to investigate the functions of cysteine in cells and diagnose diseases through its sensitive and selective detection.

Numerous techniques have been reported to monitor cysteine, including colorimetric sensor [4], HPLC methods [5], capillary electrophoresis analysis [6], spectrofluorimetry [7], electrochemical methods [8,9] and photoelectrochemical (PEC) detection [10,11]. Among the different methodologies used, photoelectrochemical measurements have attracted considerable interest because of their excellent analytical properties. In contrast to electrochemical analysis, photoelectrochemical detection exhibits a significantly reduced background as a result of the separation between the excitation light source and the detection photocurrent signal. Therefore, this method demonstrates encouraging analytical uses in the fields of bioanalysis, environmental monitoring and medicine detection [12,13]. Despite the mentioned advantages, the development and application of high-performance photoactive materials are still urgent issues to be solved in photoelectrochemical sensing research.

Transition-metal dichalcogenides (TMDs) are a family of graphene-like, two-dimensional layered materials that has attracted widespread attention in the research community [14]. Recent studies have shown that TMDs have a large specific surface area and abundant active sites, which can significantly enhance the catalytic activity of these materials [15]. As members of the TMD compound group, tungsten disulfide (WS_2_) nanosheets exhibit extraordinary properties, including less toxicity, high intrinsic conductivity and extraordinary electrical and optical performance, and thus they have been widely used in the field of photocatalysis and electrochemical detection in the past few years [16,17,18,19]. Nevertheless, the inadequate water dispersion of WS_2_ nanosheets, limited light absorption capability and rapid recombination of photoinduced electron–hole pairs hinder their further application in photoelectrochemical detection. Hence, researchers have attempted to build hybrid nanostructures using WS_2_ to enhance dispersion characteristics and photoelectrochemical performance [20,21,22,23]. Cadmium sulfide (CdS) is recognized as an attractive active material among PEC materials due to its exceptional ability to absorb visible light and ideal band gap. However, CdS alone exhibits photocorrosion and low photoelectric conversion efficiency under visible light irradiation [24]. Researchers have reported that nanocomposites exhibit a significant enhancement in photocatalytic performance when they have a large area comprising a few layers of WS_2_ combined with CdS particles [25]. However, the reported preparation method needs a high temperature, long preparation time and complex experimental conditions. Furthermore, CdS–WS_2_ hybrid materials prepared through one-pot synthesis do not easily form uniform thin films on indium tin oxide (ITO) electrodes due to their poor dispersion property. Therefore, the fabrication and application of high-performance CdS–WS_2_ heterojunction thin film remain challenging tasks in the research of photoelectrochemical sensors.

In the past few decades, numerous methods have been reported for preparing multi-metal nanomaterial films, including the use of layer-by-layer films and self-assembled monolayers, as well as various electrosynthesis techniques, which have been applied in different fields [26,27]. Among the different methodologies, electrophoretic deposition (EPD) has been widely used for depositing various materials, such as composites, polymers and inorganic nanomaterials, to form multifunctional thin films suitable for different applications due to its advantages of simplicity, good uniformity and fast deposition speed [28]. The research on zeta potential showed that WS_2_ flakes can carry charges in water/ethanol solution [29]. Therefore, WS_2_ thin films can be prepared with the EPD technique.

In this work, a novel PEC sensor (CdS/WS_2_/ITO) was developed using CdS-nanoparticle-decorated WS_2_ nanosheet heterojunction thin-film-modified ITO electrodes. A simple and secure room-temperature deposition method was used to prepare the two-dimensional WS_2_ nanosheet and CdS nanoparticle composites on ITO electrodes. Electrophoretic deposition was employed for the fabrication of WS_2_/ITO electrodes consisting of a few layers of WS_2_ nanosheets. Next, the successive ion layer adsorption and reaction (SILAR) technique was utilized to load CdS nanoparticles onto WS_2_/ITO. The CdS/WS_2_/ITO nanocomposites obtained by this simple and well-controlled technique exhibited significant adhesion to ITO substrates and stable PEC performance. In the heterostructures, CdS served as a substance for light absorption and photocurrent generation, whereas WS_2_ nanosheets served as a superb conductive matrix owing to their exceptional electron mobility. Given the advantageous presence of the heterointerface and the synergistic effect, enhanced PEC performance was ultimately achieved due to the significant facilitation of photogenerated electron migration to the electrode and the efficient strengthening of charge separation. In addition, layered WS_2_ with a large specific surface area can load more CdS nanoparticles and form a large contact area, resulting in a strong, effective interfacial electric field, which promotes the charge separation to improve the optoelectronic performance. Therefore, we developed an innovative PEC biosensing platform using CdS/WS_2_ nanocomposites which exhibited enhanced photocurrent response to cysteine oxidation. Based on the specific recognition between cysteine and CdS/WS_2_/ITO, through the specificity of Cd-S bonds, a light-driven photoelectrochemical sensor was fabricated for cysteine detection. The prepared biosensor exhibited excellent sensitivity, extensive linearity, exceptional selectivity and notable reproducibility in detecting cysteine. The photoelectrochemical sensing method was successfully employed to quantify cysteine levels in both human urine and blood serum.

## 2. Materials and Methods

### 2.1. Chemicals

XFNANO Materials Tech Co., Ltd. (Nanjing, China), supplied a WS_2_ nanosheet with a diameter ranging from 20 to 50 nm and a thickness of 1 to 8 nm. Sinopharm Chemical Reagent Co., Ltd. (Shanghai, China), provided cadmium nitrate, sodium sulfide, absolute ethanol, cysteine, lysine, histidine, phenylalanine, glutamic acid, tyrosine, sodium citrate, glucose, glutathione, ascorbic acid and uric acid (analytical grade). A 0.1 M amount of KH_2_PO_4_ and 0.1 M Na_2_HPO_4_ stock solutions were combined to prepare the phosphate buffer (PB).

### 2.2. Apparatus

A D8-Advance X-ray diffractometer (Bruker AXS, Karlsruhe, Germany), UV-2600 UV–visible spectrophotometer (Shimadzu, Kyoto, Japan), SU-8010 field emission scanning electron microscopy (Hitachi, Tokyo, Japan) and an ALPHA FT-IR infrared spectrometer (Bruker, Karlsruhe, Germany) were used to characterize the prepared nanomaterials. PEC experiments were performed on a homemade PEC system which included a 350 W xenon lamp as the irradiation source and an LK3200A electrochemical workstation (LANLIKE, Tianjin, China) for photocurrent detection. 

### 2.3. Construction of Photoelectrochemical Sensor

**Scheme 1:**
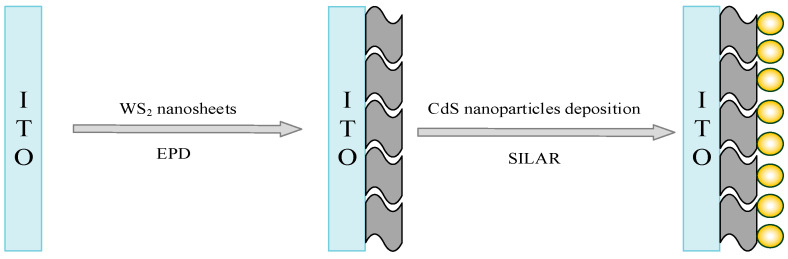
Schematic diagram of CdS/WS_2_/ITO fabricated via a coupling technique of EPD and SILAR methods.

Before its modification, the ITO slices (0.5 × 4 cm^2^) were sequentially ultrasonically cleaned in acetone and ethanol for 20 min. The photoelectrochemical sensor (CdS/WS_2_/ITO) was fabricated using two well-controlled methods after some modification based on the previous literature [30,31]. As the first step, WS_2_/ITO thin film was prepared via electrophoretic deposition. WS_2_ suspension (1 mg/mL) was prepared by dispersing WS_2_ powder in the mixed water/ethanol solution (65:35) under sonication for 30 min. Two ITO sheets arranged in parallel with a distance of 15 mm from each other were selected as anode and cathode to construct a two-electrode cell. Thus, using the prepared WS_2_ suspension as the electrolyte, deposition of WS_2_ thin film on the ITO cathode was realized by electrophoretic deposition at 8 V for 30 s. The obtained electrode is marked as WS_2_/ITO. As the second step, CdS nanoparticles were loaded on the prepared WS_2_/ITO via SILAR method. Firstly, the WS_2_/ITO electrode was immersed in a 0.05 M Cd (NO_3_)_2_ solution for 20 s, then it was immersed in a 0.05 M Na_2_S solution for 20 s. This process was repeated for 20 cycles to gain CdS/WS_2_/ITO. As a control, the same SILAR technology was employed to prepare a CdS/ITO electrode by depositing CdS nanoparticles on ITO. The preparation process of the CdS/WS_2_/ITO electrode is shown in Scheme 1.

### 2.4. Photoelectrochemical Detection of Cysteine

**Scheme 2:**
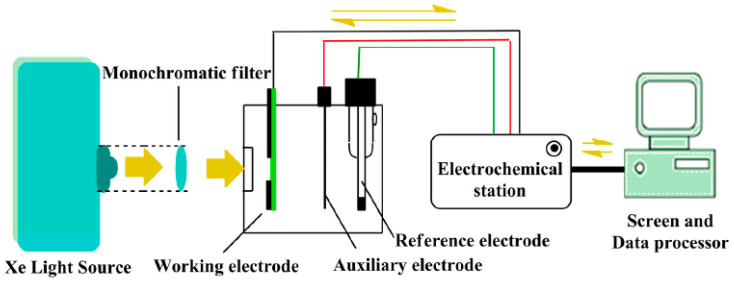
Schematic diagram of the detection process of the photoelectrochemical sensor.

As shown in Scheme 2, PEC experiments were performed on a homemade PEC system which included an irradiation source as the light excitation system and an electrochemical workstation for photocurrent detection. The detection system consists of a photoelectrochemical cell composed of a traditional electrochemical three-electrode system and a data-processing system. A three-electrode cell with a saturated calomel electrode, a platinum sheet electrode and a modified ITO electrode (0.25 cm^2^) was employed for PEC measurements. Visible light was generated by a xenon lamp with a power output of 350 W along with a UV-cut filter that blocked wavelengths below 400 nm. The distance from the working electrode to the light source remained constant at 20 cm. The current time curve method was applied to measure the photocurrent in pH 7.0 PB containing different concentrations of cysteine with an illumination interval of 20 s. During operation, the working voltage was set to 0.0 V. The experiment was conducted at ambient temperature.

## 3. Results and Discussion

### 3.1. Morphological and Spectroscopic Characterization of the Prepared Nanomaterials

**Figure 1:**
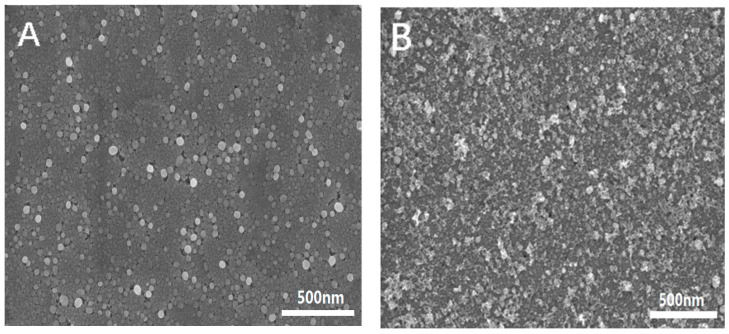
SEM image of (**A**) WS_2_/ITO and (**B**) CdS/WS_2_/ITO.

Figure 1A,B display the surface morphology of WS_2_/ITO and CdS/WS_2_/ITO at the same magnification, respectively. From the scanning electron microscopy (SEM) image in Figure 1A, it can be observed that a dense nanosheet thin film is formed on the ITO conductive glass, which is electrodeposited from a few layers of the WS_2_ nanosheets. The ITO substrate is completely covered by the WS_2_ sheets, which have a diameter ranging from 20 to 50 nm and are distributed evenly across the surface. It can also be observed that, in certain areas of Figure 1A, the bright, white protrusion structure of the WS_2_ nanosheets is formed due to sufficient WS_2_ deposition on the surface. The sufficient deposition amount of WS_2_ nanosheets increases the surface roughness of the modified electrode. The SEM image of CdS/WS_2_/ITO in Figure 1B exhibits the uniform deposition of CdS nanoparticles on the entire thin film of the WS_2_ nanosheets via the SILAR method. The CdS nanoparticles are attached and loaded on the surface of WS_2_/ITO to form a well-distributed coating layer, suggesting the blending of CdS particles into WS_2_ sheets to form a stable CdS-WS_2_ heterostructure. In addition, SEM-EDS was performed to confirm the composition of the obtained nanohybrids. The EDS spectra and atomic percentages of the elements (Figure S1) reveal the presence of Cd, S and W elements, indicating that the CdS/WS_2_/ITO film contains stoichiometric WS_2_ sheets and CdS nanoparticles. 

**Figure 2:**
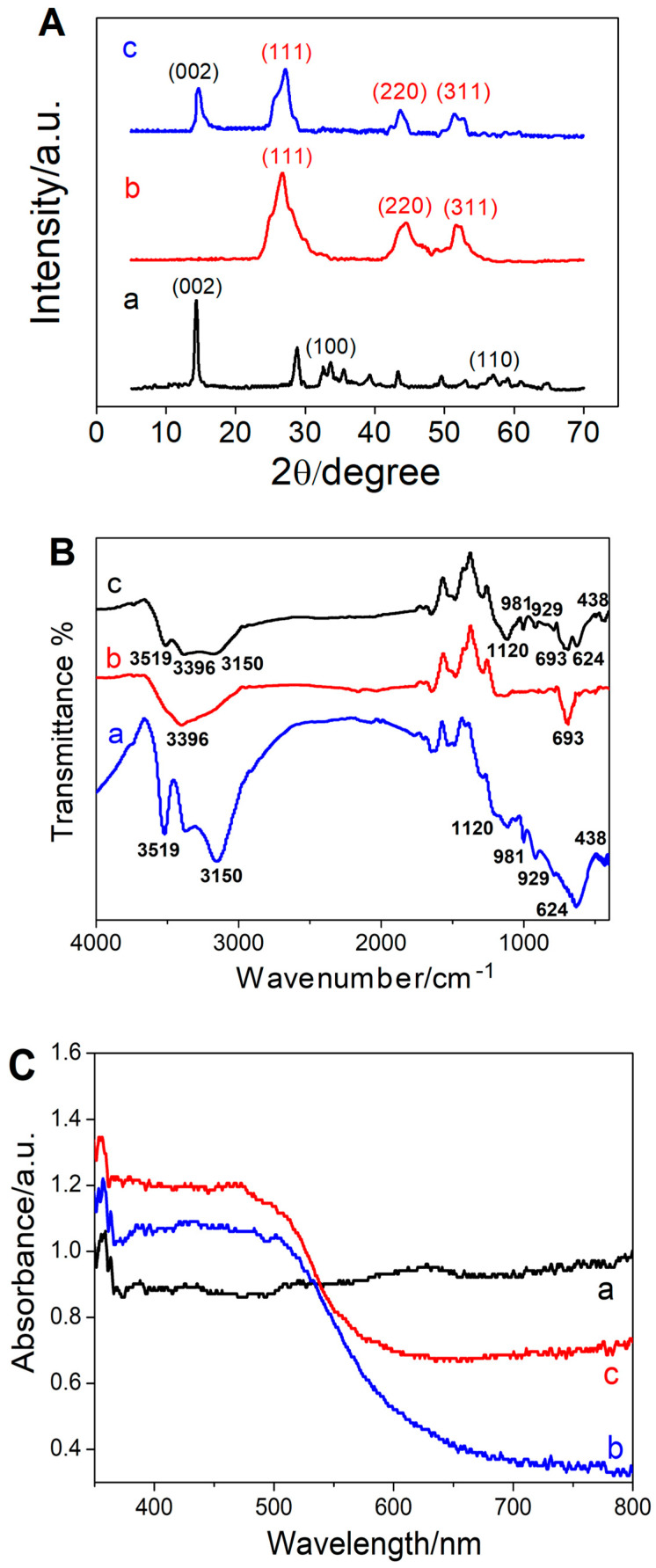

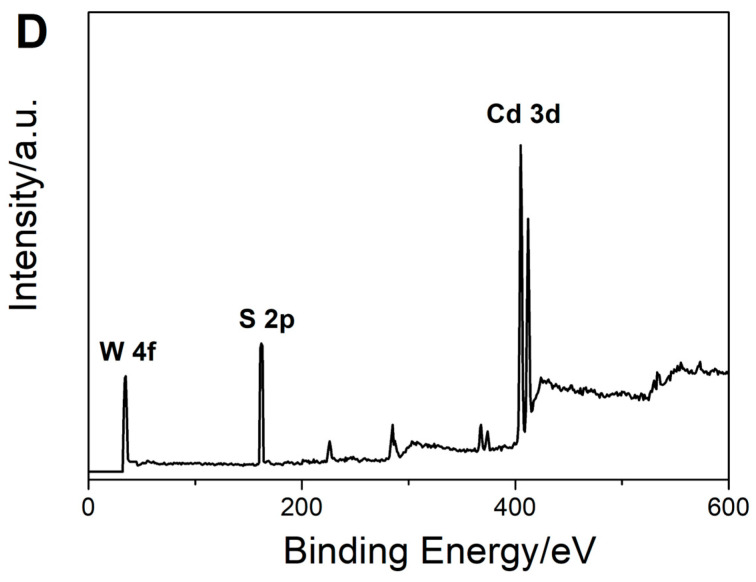
(**A**) XRD patterns, (**B**) FTIR spectra and (**C**) DRS spectra of (a) WS_2_, (b) CdS and (c) CdS-WS_2_. (**D**) XPS spectra of CdS-WS_2_.

The X-ray diffraction technique was employed to investigate the composition and structure. The XRD results in Figure 2A were collected on CdS/WS_2_ nanocomposites scraped from CdS/WS_2_/ITO using a sharp blade. The crystal structure of the WS_2_ nanosheets (Figure 2A, curve a) shows the characteristic diffraction peaks of WS_2_ at 14.4°, 28.9°, 32.5°, 33.7°, 35.4°, 39.2°, 43.4°, 49.6°, 53.2°, 57.2°, 59.2°, 61.0° and 65.1°, which are related to the (002), (004), (100), (101), (102), (103), (006), (105), (106), (110), (008), (112) and (114) planes (JCPDS no. 87-2417) [32]. Among them, the diffraction peak corresponding to the (002) crystal plane represents the vertical stacking of multilayer materials along the c-axis. The two peaks corresponding to (100) and (110) indicate that WS_2_ has a two-dimensional nanosheet structure [33], which is consistent with the morphology of WS_2_ material. The XRD pattern of CdS (Figure 2A curve b) shows three diffraction peaks at 26.5°, 44.2° and 51.8°, indicating that the CdS nanoparticles have a cubic phase structure. These peaks correspond to the (111), (220) and (311) crystal planes of cubic CdS (JCPDS no. 10-0454) [34]. It can also be observed that the peaks related to cubic CdS are relatively broad, indicating that the size of CdS nanoparticles is very small. In contrast, the XRD spectrum of CdS/WS_2_ composite material exhibits all the diffraction peaks of pure CdS and an additional peak at 14.4° (Figure 2A, curve c). This peak corresponds to the (002) crystal plane of WS_2_ [32], suggesting the successful loading of CdS nanoparticles on WS_2_ nanosheets. However, no diffraction peaks at other positions of WS_2_ are observed in curve c, indicating that these small diffraction peaks of WS_2_ are masked when combined with CdS. The above result further proves that the WS_2_ nanosheets were covered with CdS nanoparticles to form CdS/WS_2_ nanocomposites.

Figure 2B shows the FT-IR spectra of the WS_2_ nanosheets, CdS nanoparticles and CdS-WS_2_ heterojunction. In curve a, pristine WS_2_ displays its characteristic absorption peaks at 438, 624, 929, 981 and 1120 cm^−1^, corresponding to the stretching vibrations of W-S bonds and S-S bonds [35]. For CdS, the infrared absorption peak at 693 cm^−1^ is attributed to the stretching vibration of CdS (curve b) [34]. Both WS_2_ and CdS have strong absorption peaks around the range of 3200–3550 cm^−1^, which is the stretching vibration of –OH in water molecules [36]. By comparison, both the infrared characteristic absorption peaks of WS_2_ and CdS appear in curve c, which confirms the successful attachment of CdS to the surface of WS_2_.

UV–Vis diffuse reflectance spectroscopy (DRS) was applied for optical performance analysis. The DRS results in Figure 2C demonstrate that all samples had light absorption in the visible light range. The bandgap absorption edge of CdS was at 610 nm (curve b), while WS_2_ exhibited almost complete absorption in the visible region (curve a) [37]. The UV–Vis absorption spectrum of WS_2_ nanosheets in a mixture of water and ethanol is shown in Figure S2. The spectra curve further proves that WS_2_ exhibited a significant absorption in the visible light region with an absorption maximum at 640 nm [38], which is consistent with the results obtained by the UV–Vis diffuse reflectance spectroscopy (curve a). After modification of the CdS nanoparticles, the optical absorption edge of CdS-WS_2_ nanocomposites was red-shifted to 650 nm. Moreover, in the whole visible region, the heterojunction showed stronger absorption intensity compared to pure CdS (curve c). The energy bandgap (Eg) was calculated from Tauc plot. The Eg values were 1.83 eV for bare WS_2_, 2.42 eV for bare CdS and 2.26 eV for CdS-WS_2_ nanocomposites, which are consistent with the results reported in the literature [21,30]. The above results reveal that the composite material possesses a higher utilization rate of visible light and outstanding optical properties, which improves its photocatalytic ability.

The elemental composition of CdS-WS_2_ nanocomposite was evaluated using X-ray photoelectron spectroscopy (XPS). The XPS spectrum of CdS-WS_2_ in Figure 2D confirms the presence of W, S and Cd elements in the CdS-WS_2_ hybrid, which is completely consistent with the results of the FT-IR spectroscopy, indicating the successful preparation of CdS-WS_2_ nanocomposites.

### 3.2. Photoelectrochemical Performance of the Sensor

**Figure 3:**
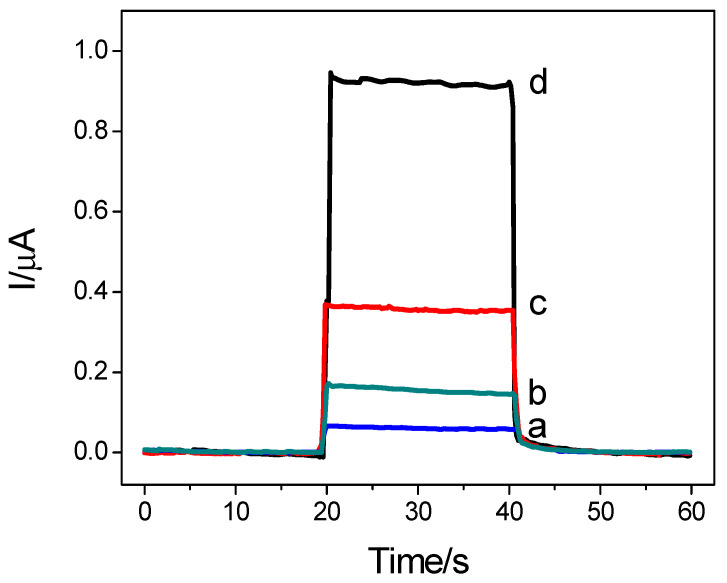
Photocurrent responses of (a) WS_2_/ITO, (b) CdS/ITO, (c) CdS/WS_2_/ITO in 0.1 M phosphate buffer (pH 7.0) and (d) CdS/WS_2_/ITO towards 10 μM cysteine in 0.1 M phosphate buffer at an applied potential of 0.0 V under the visible light irradiation.

The PEC properties of the prepared sensor were investigated by measuring the photocurrent on different modified electrodes at a working voltage of 0.0 V under visible light illumination. Figure 3 shows that WS_2_/ITO and CdS/ITO had a weak photocurrent of 0.06 μA (curve a) and 0.16 μA (curve b), respectively, which can be attributed to the fact that the single photosensitive material exhibits insufficient separation of electrons and holes and the high recombination rate of photogenerated charges under visible light irradiation. In a great comparison, the CdS/WS_2_/ITO electrode produced an obvious ascended photocurrent of 0.35 μA (curve c), which was an approximately 6-fold increase compared to that of WS_2_/ITO and a 2-fold increase compared to that of CdS/ITO. This demonstrates that the CdS-WS_2_ heterostructure can effectively inhibit the recombination of charge carriers, resulting in significantly improved photoelectric conversion efficiency and enhanced photoelectrochemical activity. As seen in curve d, the CdS/WS_2_/ITO electrode showed an enhanced photocurrent of 0.93 μA when 10 μM cysteine was present, indicating that cysteine as an electron donor can effectively promote the separation of electron–hole pairs, which leads to further ascent of the photocurrent response.

The PEC activities of the nanostructures are greatly influenced by their electrical characteristics. Electrochemical impedance spectroscopy (EIS) was conducted to investigate the interface properties of electrodes. The corresponding data and figures are provided in the Supplementary Materials. All of the EIS data (Figure S3) are in good agreement with the photocurrent change tendency in the current–time curves described above, further demonstrating that the sensing interface had been successfully constructed.

**Scheme 3:**
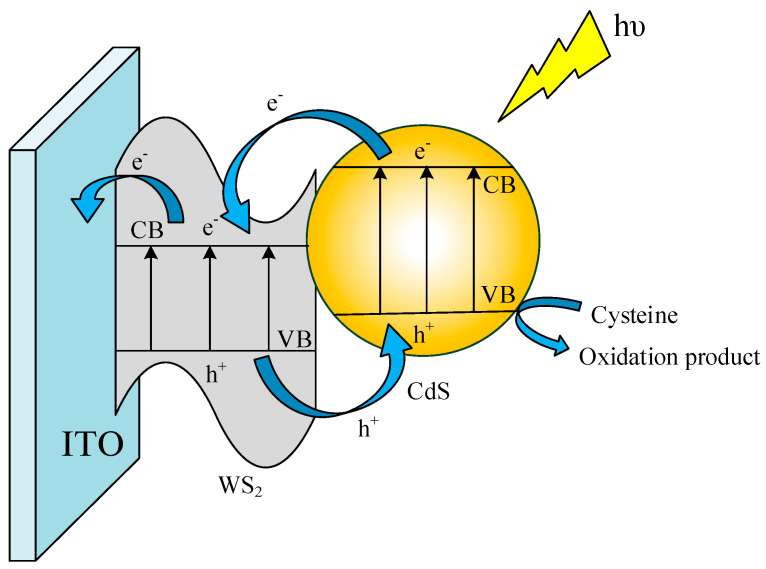
The charge transport pathway and detection principle of PEC cysteine sensor.

A schematic diagram of CdS/WS_2_/ITO for cysteine detection is presented in Scheme 3. Under visible light illumination, both CdS and WS_2_ can produce photogenerated charges. During the conversion of photocurrents, the electrons on the valence band (VB) of CdS are firstly excited to the conduction band (CB) of CdS to generate electron–hole pairs after absorbing visible light. Afterwards, the excitation electrons on the CB of CdS swiftly transfer to the CB of WS_2_ due to the presence of a heterointerface and well-matched energy levels. Together with the photogenerated electrons generated inside WS_2_, electrons on the CB of WS_2_ subsequently inject into the ITO electrode for the formation of a current in the external circuit. The separated holes on the VB of WS_2_ can transfer to the VB of CdS nanoparticles owing to energy level matching. Then, electron donor cysteine in PB captures the separated holes on the VB of CdS, resulting in the oxidization of cysteine. The consumption of the photogenerated holes by cysteine effectively suppresses charge recombination, leading to an enhanced photocurrent response in the existence of cysteine. The improvement of the photoelectrochemical properties of the PEC sensor is attributed to several factors as follows: (1) The formation of the surface heterojunctions and the effective interfacial electric field in CdS-WS_2_ nanocomposites can promote the charge separation and slow down the electron–hole recombination. This heterojunction architecture can protect CdS against photocorrosion, leading to a stable photocurrent. (2) The ITO electrode modified with the thin WS_2_ layer enhances the surface roughness, allowing for increased loading of CdS particles and more uniform coverage. Compared to CdS without a WS_2_ layer, this results in an improved light absorption capacity and more oxidation sites. (3) The WS_2_ nanosheet has excellent conductivity which can bring about rapid charge transport. The mechanism of charge transfer further confirms that the CdS-WS_2_ heterostructure exhibits distinctly enhanced PEC activity, contributing to a higher separation efficiency of photoexcited charges, faster migration rate of electrons and an excellent synergistic effect.

### 3.3. Optimization of Experimental Conditions

Various experimental parameters were optimized to enhance PEC response and improve detection sensitivity, including electrophoretic deposition time of WS_2_, SILAR deposition cycles of CdS nanoparticles and applied potential. The corresponding data and figures are provided in the Supplementary Materials (Figure S4). The best results were obtained under the following experimental conditions: (a) electrophoretic deposition time: 30 s; (b) SILAR deposition cycles: 20 cycles; (c) applied potential: 0.0 V.

### 3.4. Photoelectrochemical Sensing of Cysteine

**Figure 4:**
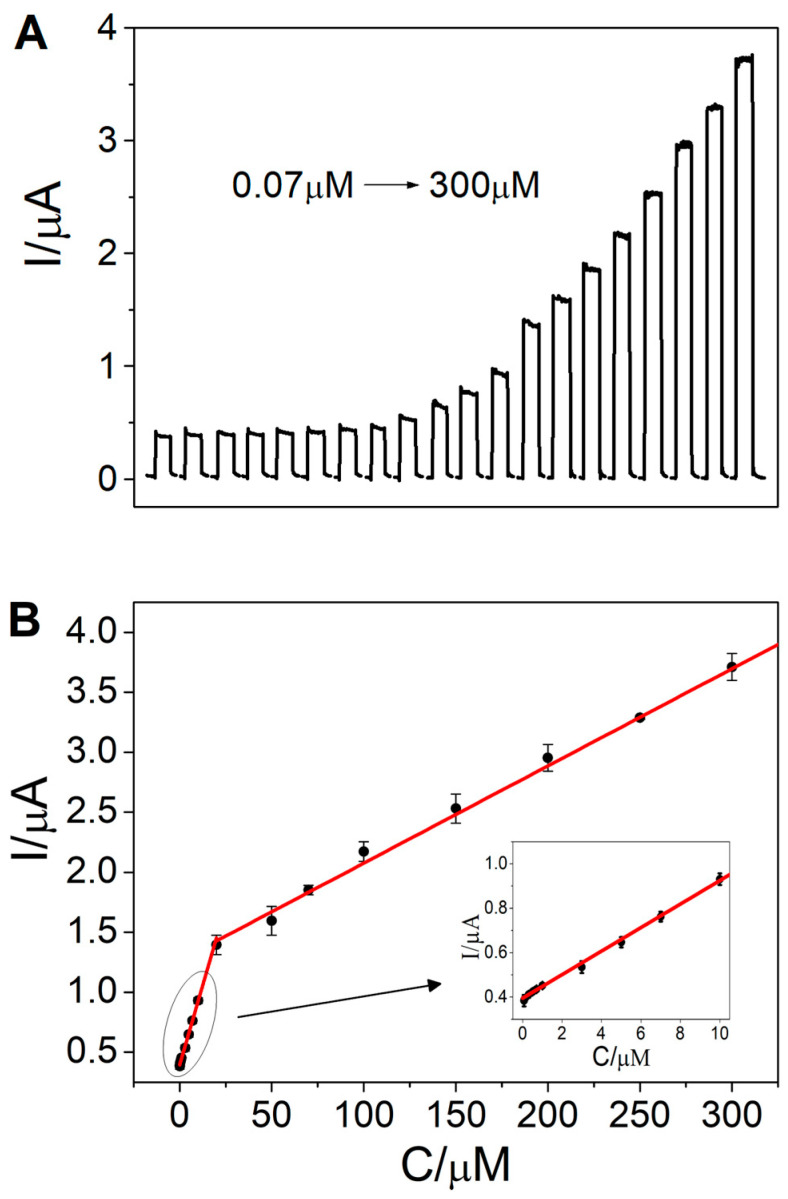
(**A**) Photocurrent response to cysteine at a series of concentrations: 0.07, 0.08, 0.09, 0.1, 0.3, 0.5, 0.7, 1.0, 3.0, 5.0, 7.0, 10, 30, 50, 70, 100, 150, 200, 250, 300 μM. (**B**) The linear calibration plot between photocurrent and cysteine concentration. Inset: magnified response of 0.07, 0.08, 0.09, 0.1, 0.3, 0.5, 0.7, 1.0, 3.0, 5.0, 7.0, 10 μM cysteine.

Figure 4A illustrates the photocurrent responses of the PEC sensor in different concentrations of cysteine under the optimum conditions, in which the photocurrent gradually increased along with the concentration. The sensor had a linear response in the 0.07 to 300 μM cysteine concentration range with a detection limit of 5.29 nM based on 3σ/S. As shown in Figure 4B, the regression equation was I (μA) = 0.3954 + 0.05294 c (μM) (R = 0.9973) in the concentration range of 0.07–20 μM and I (μA) = 1.2659 + 0.0081c (μM) (R = 0.9983) in the concentration range of 20–300 μM. In comparison to previously reported PEC methods, the PEC sensor described in the present work possesses a wider linear response range and higher sensitivity, demonstrating its exceptional analytical performance in detecting cysteine (Table 1). Furthermore, the proposed PEC sensor showed the outstanding advantages of simple structure, convenient operation, economy and fast detection.

### 3.5. Reproducibility, Stability and Selectivity

**Figure 5:**
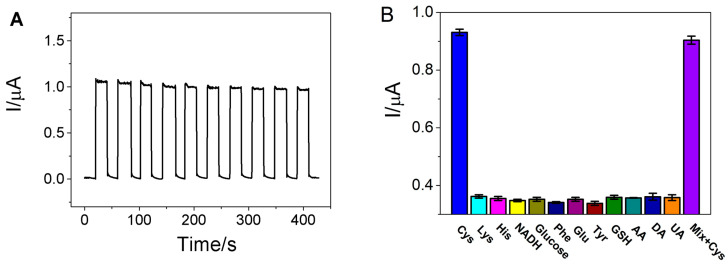
(**A**) Time-based photocurrent response of the sensor under ten on/off irradiation cycles. (**B**) Photocurrent responses of the sensor towards 10 μM of cysteine and 50 μM of other interference substances.

To assess the reproducibility, six sensors were prepared independently and applied for detecting cysteine at a concentration of 10 μM. The present evaluation yielded an RSD of 4.34%, giving favorable repeatability. To examine the stability of the sensor, the photocurrent responses of the same concentration of cysteine on the same sensor were measured 10 times (Figure 5A). The RSD of 2.89% suggests that the current response has good stability. This was attributed to the fact that the CdS-WS_2_ heterostructure can protect CdS against photocorrosion to achieve a stable photocurrent. To test the reusability of the sensor, 10 μM cysteine was measured by the same sensor ten times. The RSD of the photocurrent was 3.31%, indicating that the PEC sensor has desirable reusability. 

An interference experiment was conducted to evaluate the specificity of the sensor towards its target analyte. As displayed in Figure 5B, eleven common interferences, lysine (Lys), histidine (His), phenylalanine (Phe), tyrosine (Tyr), glutamic acid (Glu), ascorbic acid (AA), uric acid (UA), glucose, dopamine (DA), glutathione (GSH) and nicotinamide adenine dinucleotide (NADH), had no significant influence on the photocurrent response of cysteine. The mixed solution of all interfering substances and cysteine brough out a negligible photocurrent change compared to the detection signal generated by cysteine alone. The above results suggest that the proposed method has excellent selectivity for detecting cysteine owing to the specific recognition between cysteine and CdS/WS_2_/ITO through the specificity of Cd-S bonds.

### 3.6. Application in Real Sample Analysis

To assess the feasibility of the PEC sensor in detecting actual samples, the amount of cysteine in human urine and serum was measured. Human serum samples collected from a community hospital were diluted by 30 fold for detection of the photocurrent response of the PEC sensor. Human urine samples obtained from two healthy volunteers were immediately detected after a 20-fold sample dilution. The standard addition technique was used for quantitative analysis. The results in Table 2 display that the average recoveries varied from 98.3 to 102.1%, demonstrating that the developed strategy can be successfully used for complex real sample analysis. The cysteine concentrations in urine and serum detected via this method were both reasonably consistent with the results reported in the literature [46], definitely indicating the method’s practicability in clinical fluids detection.

## 4. Conclusions

In summary, on the basis of a CdS-WS_2_ heterojunction thin-film-modified ITO electrode, we constructed a novel photoelectrochemical biosensor with high sensitivity for cysteine detection. The heterostructure of CdS-nanoparticle-loaded 2D WS_2_ nanosheets can form a large contact area, strong effective interfacial electric field and synergistic effect, which promotes the charge separation to improve photoelectrochemical performance. The specific recognition between cysteine and CdS/WS_2_/ITO, through the specificity of Cd-S bonds, enhances the selectivity for cysteine detection. We afforded a simple, fast, selective and ultrasensitive photoelectrochemical technique for sensing trace cysteine in clinical detection and biological research.

## Figures and Tables

**Table 1:** Comparison of cysteine assay by various reported PEC sensors.

Working Electrode	Linear Range (μM)	Detection Limit (nM)	Reference
Nafion/CdS-MV/ITO	0.2–2.8	100	[10]
Au-SnO_2_/CdS/ITO	0.4–120	100	[11]
PTh/TiO_2_/FTO	10–800	12,800	[39]
CuO–Cu_2_O/GCE	0.2–10	50	[40]
CA-TiO_2_/FTO	2.0–100	650	[41]
ZnTAPc-Gr/ITO	0.25–113	11.4	[42]
ITO/g-C_3_N_4_/Au	10–40	9200	[43]
Bi_2_MoO_6_/TiO_2_	0.5–600	150	[44]
TiO_2_-Au-BiOI	0.8–200	70	[45]
CdS/WS_2_/ITO	0.07–300	5.29	This work

**Table 2:** Real sample analysis and recovery rate test in human serum and urine samples (n = 6).

Sample	Original (µM)	Added (µM)	Found (µM)	Recovery (%)	RSD (%)
Serum 1	3.86	5.00	8.99	101.5	2.6
Serum 2	3.95	5.00	8.83	98.7	1.8
Urine 1	4.81	5.00	9.64	98.3	3.0
Urine 2	5.56	5.00	10.78	102.1	2.3

## Data Availability

Data are contained within the article.

## Supplementary Materials

The following supporting information can be downloaded at: https://www.mdpi.com/article/10.3390/nano14050427/s1, Figure S1: EDS characterization of CdS/WS_2_/ITO. Figure S2: The UV–Vis absorption spectrum of WS_2_ nanosheets in water:ethanol (65:35) solution. Figure S3: EIS spectra of different modified electrodes: (a) WS_2_/ITO, (b) CdS/ITO, (c) CdS/WS_2_/ITO. Figure S4: (A) effect of electrophoretic deposition time of WS_2_: (a) 30s, (b) 1 min and (c) 2 min, (B) effect of SILAR cycles of CdS: (a) 5, (b) 10, (c) 15, (d) 20, (e) 25 and (f) 30 cycles, (C) influence of applied potential. The PEC measurements were carried out in 0.1 M PB (pH 7.0) containing 10 μM cysteine under the visible light irradiation.

## Abbreviations

TMDs, transition-metal dichalcogenides; PEC, photoelectrochemical; WS_2_, tungsten disulfide; ITO, indium tin oxide; CdS, cadmium sulfide; Eg, energy bandgap; VB, valence band; CB, conduction band; EIS, electrochemical impedance spectroscopy; Lys, lysine; His, histidine; Phe, phenylalanine; Tyr, tyrosine; Glu, glutamic acid; AA, ascorbic acid; UA, uric acid; DA, dopamine; GSH, glutathione; NADH, nicotinamide adenine dinucleotide.
