# Supplementary material for: A Photoelectrochemical Sensor for the Sensitive Detection of Cysteine Based on Cadmium Sulfide/Tungsten Disulfide Nanocomposites
Source: Nanomaterials (Basel). 2024 Feb 27;14(5):427. doi: 10.3390/nano14050427 (PMC10935151; doi:10.3390/nano14050427)
Supplement: Supplementary file 1 [file nanomaterials-14-00427-s001.zip › nanomaterials-2860746-supplementary.pdf]

Supplementary Materials

A Photoelectrochemical Sensor for the Sensitive Detection of Cysteine Based on Cadmium Sulfide/Tungsten Disulfide Nanocomposites

Yan Wang *, Jiaxin Liu and Fancheng Lin

College of Chemistry, Chemical Engineering and Materials Science, Shandong Normal University, Jinan 250014, China; sdnuljx@163.com (J.L.); sdnulfc@163.com (F.L.)

* Correspondence: fagong@sdu.edu.cn; Tel.: +86-531-89212269

1. EDS characterization of $CdS/WS_2/ITO$

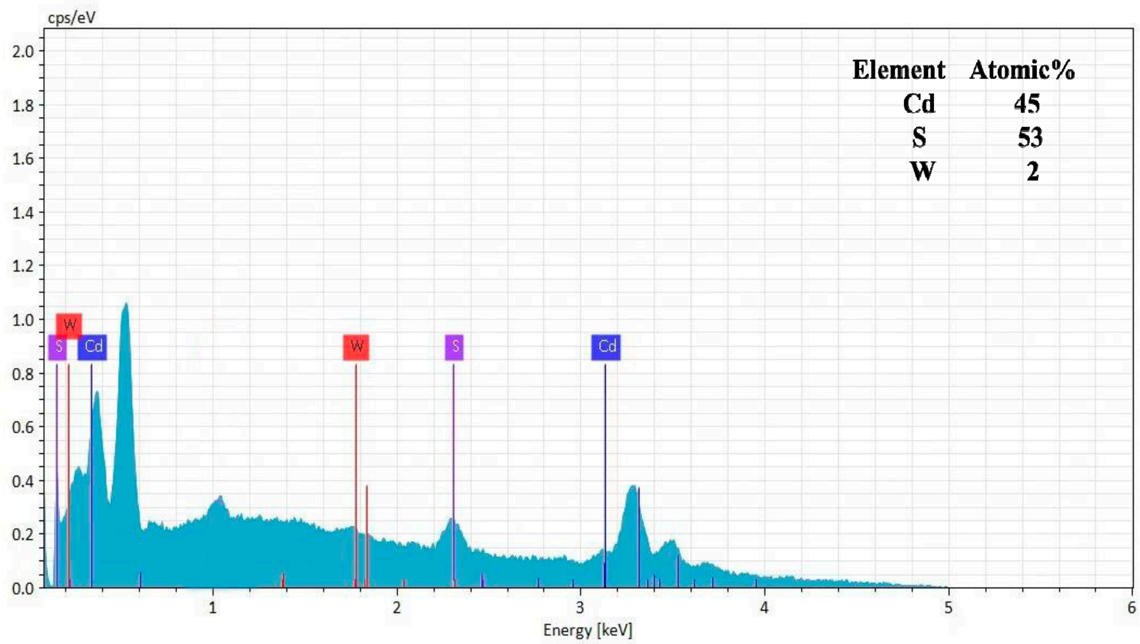

Figure S1. EDS characterization of $CdS/WS_2/ITO$.

2. The UV-Vis absorption spectrum of WS_2 nanosheets

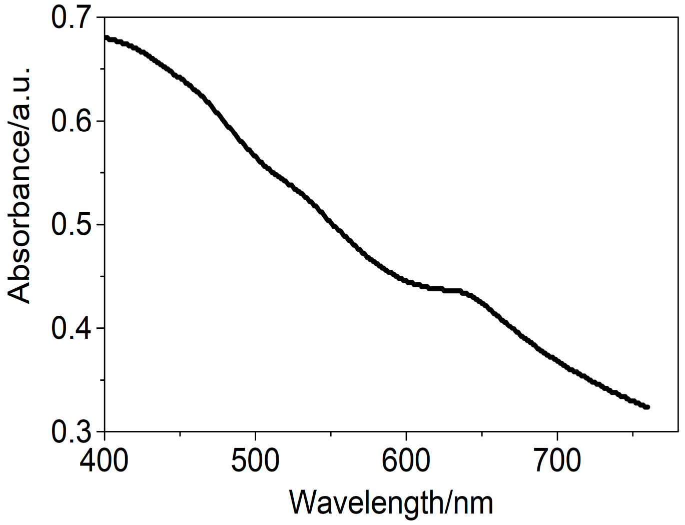

Figure S2. The UV-Vis absorption spectrum of WS_2 nanosheets in water: ethanol (65:35) solution.

3. EIS spectroscopy results

As an effective tool for characterizing the interface properties of electrodes, electrochemical impedance spectroscopy (EIS) was used to investigate the carrier transport properties at the semiconductor electrolyte interface. The measurements were carried out in 0.1 M KCl solution containing 5.0 mM $K_3Fe\ (CN)_6/K_4Fe\ (CN)_6$ (1:1). EIS was recorded in the frequency range of 0.1 Hz to 100 kHz with amplitude of 10 mV. Nyquist plots of the WS_2/ITO, CdS/ITO and CdS/WS_2/ITO thin films are shown in Figure S1. The semicircle diameter at higher frequencies corresponds to the electron-transfer resistance (R_{et}), which reflects the restricted diffusion of the redox probe through the multilayer system related directly to film permeability. The linear portion at lower frequencies corresponds to diffusion-limited process. As shown in Figure S3, Nyquist plot for pristine WS_2/ITO electrode exhibited a small semicircle diameter (curve a), indicating low R_{et} of electrode due to high intrinsic conductivity of WS_2 nanosheets. After modified with CdS, the resistance R_{et} increased (curve c) because the semiconductor property of the CdS with low conductivity led to the increase of the impedance. In comparison, the R_{et} value of bare CdS/ITO thin film (curve b) is larger than that of CdS/WS_2/ITO, which was attributed to the excellent charge transport efficiency of WS_2 to enhance electronic conductivity of CdS-WS_2 heterojunction nanocomposites. The above EIS results indicated that the PEC sensor had been successfully fabricated.

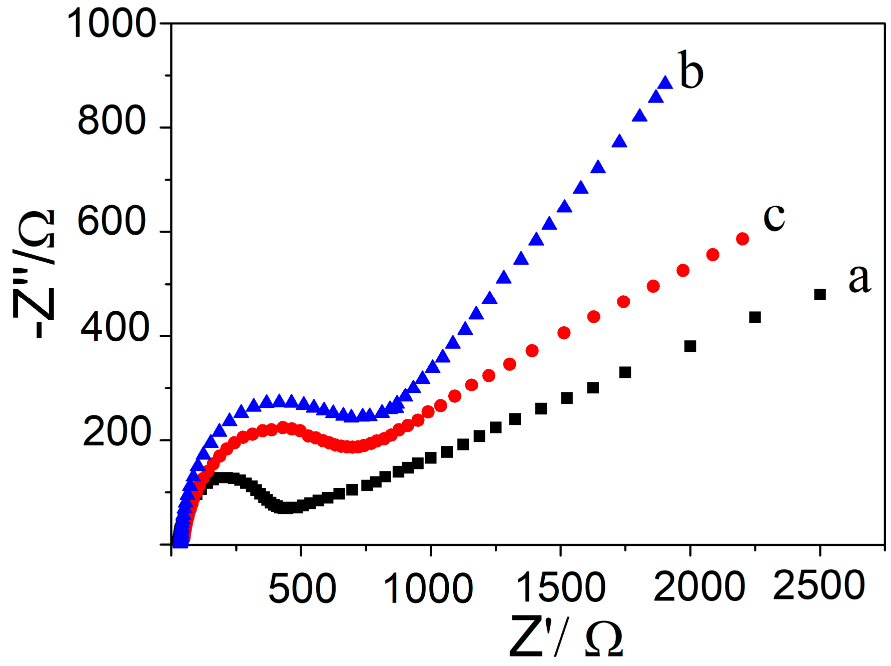

Figure S3. EIS spectra of different modified electrode. (a) WS_2/ITO, (b) CdS/ITO, (c) CdS/WS_2/ITO.

4. Optimization of experimental conditions for PEC detection

4.1. Effect of electrophoretic deposition time

In order to obtain the optimal performance of CdS/WS_2/ITO electrode for PEC detection, the effect of the electrophoretic deposition time of WS_2 was studied. As shown in Fig. S4A, the photocurrent of cysteine decreased with increasing electrophoretic deposition time from 30 seconds to 2 minutes. During electrophoretic deposition, the highest surface roughness was obtained by short deposition time and after that the surface roughness decreased and reached to a saturated value. The appropriate thickness of WS_2 nanosheets can provide larger surface roughness and more contact sites for the attachment of CdS nanoparticles. When the electrophoretic deposition time was higher than 30 seconds, the prepared WS_2 nanosheet thin layer gradually increased in deposition amount, leading to a smoother electrode surface. The surface roughness of the electrode was relatively small, which was not conducive to the deposition of CdS nanoparticles. Since the electrophoretic deposition time lower than 30 s resulted in no significant WS_2 deposition, 30 s was selected as optimized deposition time.

4.2. Effect of SILAR deposition cycles

As the deposition amount of CdS nanoparticles on WS_2 nanosheet thin layer has significant effects on the PEC performance of the prepared sensor, the effect of SILAR deposition cycles of CdS was investigated. Fig. S4B displayed that there was a gradual increase in the photocurrent of cysteine with increasing SILAR deposition number from 5 to 20 cycles. However, the current response decreased slowly when the number of deposition cycles increased from 20 to 30, which could be attributed to cluster structures formed by the excessive deposition of CdS nanoparticles that impeded the transfer of photogenerated

electrons to the WS_2 layer, leading to increase electron hole recombination and reduce the photocurrent response of the sensor. Thus, 20 cycles were selected as the optimal number of SILAR deposition in this research.

4.3. Influence of applied potential

The applied detection potential is a vital influence factor for the sensitivity of the sensor. Fig. S4C showed the effect of the detection potential on the cysteine detection. The photocurrent intensity increased with the increase of voltage in the range of -0.3~0.0 V. However, the trend of photocurrent changes with potential tended to be gentle after 0.0 V. In order to eliminate the interference of some coexisting reducing substances, a lower potential is more suitable for photoelectric detection of cysteine. Therefore, 0.0 V was chosen to be the optimized applied potential to ensure the sensitivity and selectivity of detection.

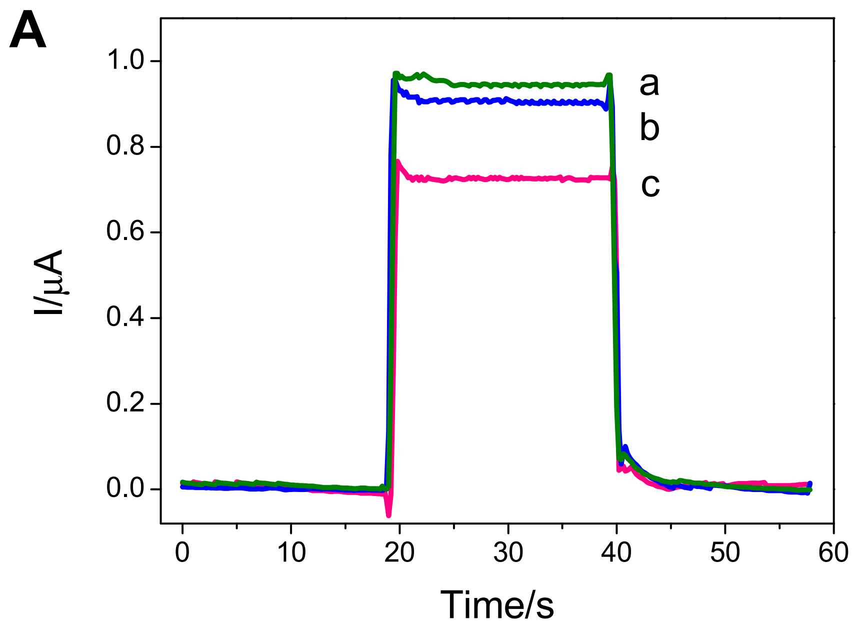

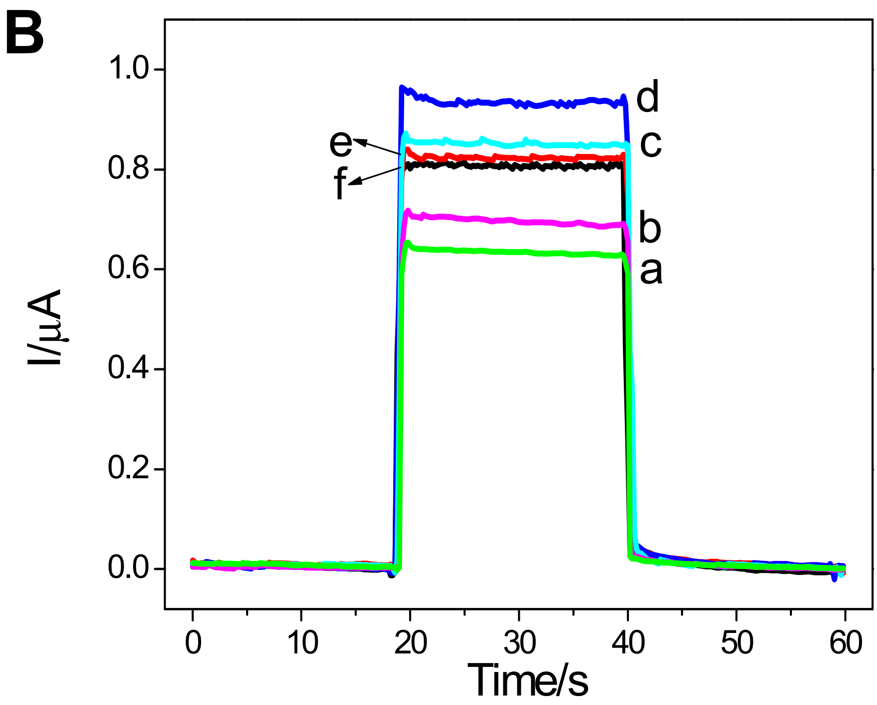

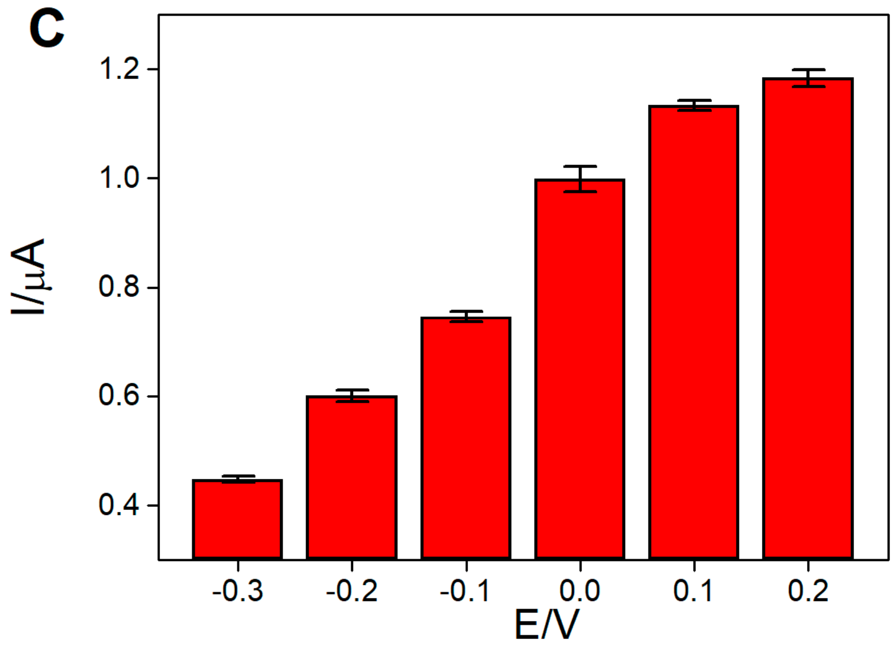

Figure S4. (A) Effect of electrophoretic deposition time of WS_2: (a) 30s, (b) 1min and (c) 2min; (B) Effect of SILAR cycles of CdS: (a) 5, (b) 10, (c) 15, (d) 20, (e) 25 and (f) 30 cycles. (C) Influence of applied potential. The PEC measurements were carried out in 0.1 M PB (pH 7.0) containing 10 μM cysteine under the visible light irradiation.
